# Public Awareness, Knowledge, and Attitude Toward Alzheimer’s Disease in Makkah, Saudi Arabia

**DOI:** 10.7759/cureus.49047

**Published:** 2023-11-19

**Authors:** Jihad Muglan, Reem M Alkhaldi, Manar M Alsharif, Shatha I Almuwallad, Rahaf S Alotaibi

**Affiliations:** 1 Department of Medicine, College of Medicine, Umm Al-Qura University, Makkah, SAU; 2 College of Medicine, Umm Al-Qura University, Makkah, SAU

**Keywords:** knowledge, level of awareness, makkah, saudi arabia, alzheimer’s disease

## Abstract

Background: Alzheimer's disease is a neurodegenerative disease that slowly deteriorates cognitive function over time. This condition disables the geriatric population worldwide. Knowing its symptoms and presentation could help the general population seek medical attention early.

Objective: This study aims to assess the level of awareness, knowledge, and attitude towards Alzheimer’s disease among the general population in Makkah City.

Methods: This cross-sectional study employed an online questionnaire distributed randomly in Makkah, Saudi Arabia. A sociodemographic and attitude panel is included under each section of the questionnaire, as well as a knowledge panel based on the Alzheimer's Disease Knowledge Scale (ADKS). The knowledge and awareness level regarding Alzheimer’s disease was determined by adding up discrete scores for each correct knowledge item. A participant's awareness level was categorized as poor if their score was less than 60%. Participants whose scores were 60% or higher were considered to have a high level of awareness

Results: A total of 545 participants were investigated; 316 (58%) were females. A range of ages was represented among the participants, from 18 to over 60. Of the study respondents, 68 (12.5%) had an overall good awareness and knowledge of Alzheimer’s disease and its management while 477 (87.5%) had a poor knowledge level. Among divorced/widowed participants, 16.2% had an overall good knowledge level of the disease compared to 8.3% of married respondents with recorded statistical significance (P=.049). Also, 20.4% of those with relatives diagnosed with Alzheimer's disease had good knowledge of the disease versus 10.7% of others without (P=.009).

Conclusion: According to the results, there is a lack of awareness and knowledge of Alzheimer’s disease. This study suggests increasing public awareness and knowledge of Alzheimer’s disease through campaigns and public education so that the disease is detected earlier.

## Introduction

Alzheimer’s disease is the most common form of dementia worldwide. It can be defined as a neurodegenerative disease characterized by slowly progressive loss of cognitive functions such as memory, thinking skills, and behavioral changes [1]. There are two main types of Alzheimer's: Early-onset Alzheimer's, which affects those in their 40s to 50s, and late-onset Alzheimer's, which affects those over 65 years of age. The Saudi Alzheimer's Disease Association estimates that 130 thousand people suffer from Alzheimer's disease in Saudi Arabia. The number of patients worldwide will double by 2050 [2].

Alzheimer’s disease affects daily living activities such as feeding, toileting, and the simplest tasks. It also affects patients' families and causes an economic burden with estimated costs of US$1 trillion globally [3].

According to studies by Connell et al., perceptions and beliefs about Alzheimer's disease are very important to incorporate the recent cultural changes due to the accelerated prevalence and excess disease burden experienced by people in developed countries [4,5].

Early detection of Alzheimer's disease ensures that affected individuals and their caregivers can make appropriate plans and decisions for their healthcare needs [6]. Yet previous studies assessing attitudes and behaviors toward Alzheimer's disease in Europe showed that the general public lacks adequate information regarding the disease or the benefits of early diagnosis and treatment [7].

Despite the lack of proven prevention strategies for Alzheimer's disease, several studies have hypothesized that several protective factors, such as mental activity, regular physical exercise, and a healthy diet, are significantly associated with a decreased risk of Alzheimer's disease [8-13]. Nevertheless, little information exists about how general public attitudes and knowledge about Alzheimer's disease affect their health behaviors regarding Alzheimer's disease prevention.

According to a previous study conducted in the Aseer region, Saudi Arabia, respondents who are young, females, and have a relative with Alzheimer's disease have good knowledge of the disease [14]. However, to date, no previous research has assessed the knowledge, attitudes, and perceptions surrounding Alzheimer's disease among the general public in the Makkah region. Therefore, this study aimed to assess public awareness, knowledge, and attitudes toward Alzheimer’s disease. We also assessed socio-demographic factors influencing public knowledge of Alzheimer's disease.

## Materials and methods

Study designs 

The study was conducted on a community-based prospective basis, targeting the general population of Makkah between January 30, 2023, and February 12, 2023. We ensured participants' privacy by avoiding asking for names. Approval was obtained beforehand from the biomedical ethics committee of Umm Al-Qura University (approval number: HAPO-02-K-012-2023-01-1370).

Study population and sampling methodology

In this study, the Raosoft online sample size calculator was used to calculate a sample size of 385 based on a margin of error of 5%, a confidence level of 95%, and an estimated total population of 2,115,000. A total of 545 participants were included in the study and convenience sampling was used. All adults over the age of 18 who agreed to participate in the questionnaire and successfully completed it were eligible to participate in the study. The participants who could not meet the criteria for participation were excluded from the study. A non-probability snowball sampling technique was used as the sampling method. In order to protect the privacy of the participants, the questionnaires were filled out anonymously. We kept all data confidential and used it only for research purposes.

Data collection 

An online Arabic questionnaire was designed using Google Forms. Respondents received electronic links via social media platforms accompanied by the survey objectives, the target population, and a request to participate voluntarily. Survey responses were collected anonymously. No identifying information and no private information was collected from participants. All responses were confidentially maintained.

The Alzheimer's Disease Knowledge Scale (ADKS) was used in this study, which was previously published and reviewed [15]. A translation to Arabic was done to assure insurers of an accurate response. According to similar published work conducted in the Aseer region, Saudi Arabia, the questionnaire was edited and validated with three neurology consultants [14]. 

The questionnaire was divided into three sections. The first section covered socio-demographic data, such as age, gender, and qualification. The second part focused on responders' awareness of the disease. Multiple statements were given, and the survey taker had the choice of agreeing. In the third section, to assess knowledge, survey takers were questioned on risk factors, assessment and diagnosis, course of the disease, caregiving, treatment, and symptoms. The questionnaire is available in the Appendices.

Data analysis

Statistical Package for Social Sciences (SPSS) version 21 (IBM Corp., Armonk, NY) was used to analyze the data. All statistical methods were two-tailed with an alpha level of 0.05, considering significance if the P value is less than or equal to 0.05. Overall knowledge and awareness level regarding Alzheimer’s disease was assessed by summing up discrete scores for different correct knowledge items. If the participant's score was less than 60% of the overall score, they were considered poor in awareness. Participants with 60% or higher scores were considered to have a good awareness level. For study variables, a descriptive analysis was conducted using frequency distributions and percentages. These variables included participants' personal data, public perception and attitude toward Alzheimer’s disease, knowledge, and awareness items. In addition, the participants' overall awareness level was graphed. Cross tabulation for showing the distribution of participants’ overall knowledge and awareness level by their personal data. This is done using the Pearson chi-square test for significance and an exact probability test if there were small frequency distributions.

## Results

A total of 545 eligible participants completed the study questionnaire. They ranged in age from 18 to more than 60 years, with 531 (97.4%) aged 18-60. Exactly 316 (58%) participants were females, 304 (55.8%) were single, and 204 (37.4%) were married. As for educational level, 357 (65.5%) were university graduates, 127 (23.3%) had a secondary education, and 49 (9%) had a post-graduate degree. A total of 321 (58.9%) of the respondents lived in small families, 198 (36.3%) lived in large families, and only 26 (4.8%) lived alone. About 98 (18%) had a relative diagnosed with Alzheimer's disease (Table 1)

**Table 1:** Personal data of study participants, Makkah, Saudi Arabia

Personal data	No	%
Age in years		
18-60	531	97.4%
> 60	14	2.6%
Gender		
Male	229	42.0%
Female	316	58.0%
Marital status		
Single	304	55.8%
Married	204	37.4%
Divorced / widow	37	6.8%
Educational level		
Below secondary	12	2.2%
Secondary	127	23.3%
University	357	65.5%
Post-graduate	49	9.0%
Living status		
Alone	26	4.8%
Small family	321	58.9%
Big family	198	36.3%
Do you have a relative diagnosed with Alzheimer's disease?		
Yes	98	18.0%
No	447	82.0%

The public's attitude toward Alzheimer’s disease in Makkah, Saudi Arabia is detailed in Table 2. Exactly 63.7% of the study respondents think that with a change in daily plans, the elderly may find it difficult to balance their finances, and 60.6% agree that once an Alzheimer's patient encounters difficulty in performing daily tasks, there is a need to resort to the judiciary to preserve the patient's rights. Likewise, 52.1% of the study respondents prefer not to inform the person, once diagnosed with Alzheimer's disease, and 41.5% think that memory disturbance and forgetting are normal for the elderly and do not require medical advice. About 33.9% of the study participants agree that once they have symptoms of dementia and memory disturbances, they resort to common types of Falk medicine. In addition, 28.3% think it is better for diagnosed patients to avoid going to social events and life activities to avoid embarrassment. Only 12.1% feel embarrassed if a relative was diagnosed with Alzheimer's disease and 19.6% dismiss a diagnosis when a relative has Alzheimer's disease.

**Table 2:** Public's attitude toward Alzheimer’s disease in Makkah, Saudi Arabia

Attitude items	Agree		Disagree	
	No	%	No	%
Do you think that memory disturbance and forgetting are normal for the elderly and do not require medical advice?	226	41.5%	319	58.5%
Do you think that the change in daily plans and the difficulty of balancing financial accounts is expected in the elderly?	347	63.7%	198	36.3%
If someone in your family has been diagnosed with Alzheimer's disease, would you prefer not to tell the person with the disease?	284	52.1%	261	47.9%
If the patient is diagnosed with Alzheimer's, do you think it is better for him to avoid going to social events and life activities to avoid embarrassing the patient?	154	28.3%	391	71.7%
Alzheimer's disease can be the result of black magic or envy?	130	23.9%	415	76.1%
In the event of symptoms of dementia and memory disturbances, would you resort to common types of Falk medicine?	185	33.9%	360	66.1%
Once an Alzheimer's patient encounters difficulty in performing daily tasks, do you see the need to resort to the judiciary to preserve the patient's rights?	330	60.6%	215	39.4%
Do you feel embarrassed if your relative was diagnosed with Alzheimer's disease?	66	12.1%	479	87.9%
Do you tend to dismiss a diagnosis when a relative has Alzheimer's disease?	107	19.6%	438	80.4%
Are you in favor of caring for Alzheimer's patients in nursing homes rather than keeping them at home?	152	27.9%	393	72.1%

The public's knowledge and awareness regarding Alzheimer’s disease in Makkah, Saudi Arabia is detailed in Table 3. Regarding Alzheimer's risk factor knowledge, only 12.3% correctly report that mindfulness exercises cannot prevent a person from developing Alzheimer's disease, while 48.3% are aware that genes can only be partially responsible for causing Alzheimer's disease, 34.1% know that people at the age of 30 can develop Alzheimer's disease, and 25.3% correctly say that high blood pressure may increase a person's risk of getting Alzheimer's disease. As for Alzheimer's effect on patients' lives, 61.1% know patients with Alzheimer's disease are more likely to suffer from depression, 48.6% of respondents say it is safe for Alzheimer's patients to drive, as long as they have a companion in their car at all times, and 27% reject nursing homes for people with Alzheimer's disease. Regarding evaluation and diagnosis, 64.4% of the respondents know that Alzheimer's disease is a type of dementia, 56.7% report that when a person with Alzheimer's disease becomes agitated, a medical evaluation may reveal health problems that caused the disorder, 39.6% are aware that symptoms of severe depression can be mistaken for Alzheimer's disease, and 24.6% do not believe that if memory and confusion problems start suddenly, they are likely caused by Alzheimer's disease. Regarding Alzheimer's disease progression, 61.7% of participants know that Alzheimer's patients will require 24 hours of supervision. In addition, 49.9% of respondents reported that people with Alzheimer's will fall more often as the disease progresses. However, 16.5% say the average lifespan after Alzheimer's symptoms appear is 6 to 12 years.

**Table 3:** Public knowledge and awareness regarding Alzheimer’s disease in Makkah, Saudi Arabia

Knowledge & awareness items	Correct		Incorrect		I don’t know	
	No	%	No	%	No	%
Alzheimer risk factors						
Mindfulness exercises can prevent a person from developing Alzheimer's disease	306	56.1%	67	12.3%	172	31.6%
People at the age of 30 can develop Alzheimer's disease	186	34.1%	113	20.7%	246	45.1%
High cholesterol may increase a person's risk of developing Alzheimer's disease	121	22.2%	70	12.8%	354	65.0%
Medications that help delay the progression of Alzheimer's disease are available	262	48.1%	59	10.8%	224	41.1%
High blood pressure may increase a person's risk of developing Alzheimer's disease	138	25.3%	86	15.8%	321	58.9%
Genes can only be partially responsible for developing Alzheimer's disease	263	48.3%	82	15.0%	200	36.7%
Alzheimer effect on patient's life						
People with Alzheimer's disease are particularly prone to depression	333	61.1%	68	12.5%	144	26.4%
Most people with Alzheimer's disease live in nursing homes	182	33.4%	147	27.0%	216	39.6%
It is safe for people with Alzheimer's disease to drive, as long as they have a companion in the car at all times	183	33.6%	265	48.6%	97	17.8%
Evaluation and diagnosis						
When a person with Alzheimer's disease becomes agitated, a medical evaluation may reveal health problems that caused the disorder	309	56.7%	40	7.3%	196	36.0%
If the problem with memory and confusion started suddenly, it is most likely due to Alzheimer's disease	223	40.9%	134	24.6%	188	34.5%
Symptoms of severe depression can be mistaken for Alzheimer's disease	216	39.6%	120	22.0%	209	38.3%
Alzheimer's disease is a type of dementia	351	64.4%	71	13.0%	123	22.6%
Alzheimer disease progression						
After Alzheimer's symptoms appear, the average life expectancy is 6 to 12 years	90	16.5%	161	29.5%	294	53.9%
In rare cases, people recover from Alzheimer's disease	151	27.7%	131	24.0%	263	48.3%
A person with Alzheimer's disease is more likely to fall as the disease progresses	272	49.9%	58	10.6%	215	39.4%
Ultimately, a person with Alzheimer's disease will need 24 hours of supervision	336	61.7%	80	14.7%	129	23.7%

Table 4. Public knowledge and awareness regarding Alzheimer’s disease in Makkah, Saudi Arabia, continued. For patient care, 49.9% are aware that simple instructions are best for people with Alzheimer's disease, and 49.4% correctly report that if a person with Alzheimer's disease becomes alert and restless at night, a good strategy is to make sure that they get plenty of exercise during the day, but 8.1% deny that when Alzheimer's patients have difficulty taking care of themselves, caregivers must take matters into their own hands immediately. Regarding management, 56.7% of the study respondents know that malnutrition can exacerbate Alzheimer's symptoms, 51.8% of respondents say that people with early Alzheimer's disease can receive therapy for anxiety and depression, while 36.5% deny that reminder notes can lead to deterioration in Alzheimer's patients. As for disease signs, 40.6% know that one of the symptoms that can occur with Alzheimer's disease is the belief that other people steal things, 39.4% believe that difficulty handling money or paying bills is one of the earliest symptoms of Alzheimer's, while 21.8% know that tremor, or shaking of the hands or arms, is not a common symptom in people with Alzheimer's disease.

**Table 4:** Public knowledge and awareness regarding Alzheimer’s disease in Makkah, Saudi Arabia, continued

Knowledge & awareness items, continued	Correct		Incorrect		I don’t know	
	No	%	No	%	No	%
Patients care						
People with Alzheimer's disease do best with simple instructions given one step at a time	272	49.9%	69	12.7%	204	37.4%
When people with Alzheimer's begin to have difficulty taking care of themselves, caregivers must take matters into their own hands immediately.	382	70.1%	44	8.1%	119	21.8%
If a person with Alzheimer's disease becomes alert and restless at night, a good strategy is to try to ensure that the person gets plenty of physical activity during the day	269	49.4%	49	9.0%	227	41.7%
When people with Alzheimer's disease repeat the same question or story many times, it is helpful to remind them that they are repeating themselves.	136	25.0%	236	43.3%	173	31.7%
Once people have Alzheimer's disease, they are no longer able to make decisions about their care	258	47.3%	117	21.5%	170	31.2%
Management						
People with Alzheimer's disease that is not yet severe can benefit from psychotherapy for depression and anxiety	288	52.8%	36	6.6%	221	40.6%
Malnutrition can exacerbate Alzheimer's symptoms	309	56.7%	40	7.3%	196	36.0%
When a person has Alzheimer's disease, using reminder notes can contribute to deterioration	157	28.8%	199	36.5%	189	34.7%
Alzheimer's disease cannot be cured	196	36.0%	113	20.7%	236	43.3%
Diseases signs						
Tremor, or shaking of the hands or arms, is a common symptom in people with Alzheimer's disease	147	27.0%	119	21.8%	279	51.2%
Trouble handling money or paying bills is a common early symptom of Alzheimer's disease	215	39.4%	91	16.7%	239	43.9%
One of the symptoms that can occur with Alzheimer's disease is the belief that other people steal things	221	40.6%	69	12.7%	255	46.8%
Most people with Alzheimer's disease remember recent events better than things that happened in the past	150	27.5%	217	39.8%	178	32.7%

**Figure 1:**
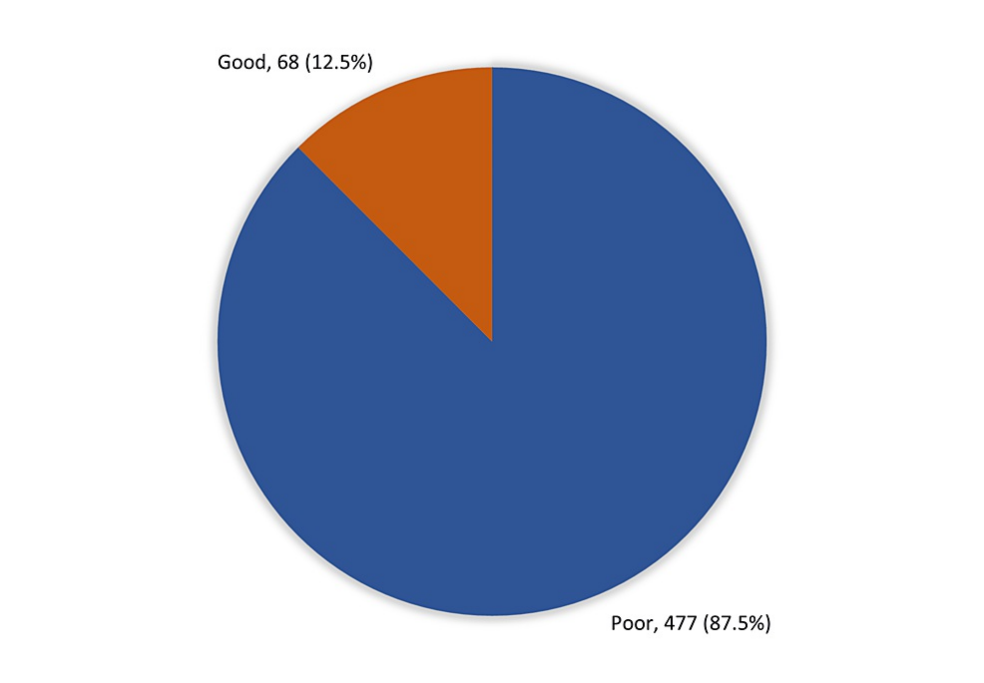
Overall participants' knowledge and awareness regarding Alzheimer’s disease in Makkah, Saudi Arabia

Overall participants' knowledge and awareness regarding Alzheimer’s disease in Makkah, Saudi Arabia is shown in Figure 1. Among the study respondents, 68 (12.5%) had good knowledge and awareness of Alzheimer's disease while 477 (87.5%) had poor knowledge.

Factors associated with participants' knowledge and awareness regarding Alzheimer’s disease are detailed in Table 5. Exact 16.2% of divorced /widowed participants had an overall good knowledge level of the disease compared to 8.3% of married respondents with recorded statistical significance (P=.049). Also, 20.4% of those with relatives diagnosed with Alzheimer's disease had good knowledge regarding the disease versus 10.7% of others without (P=.009). All other factors were insignificantly associated with participants' knowledge level.

**Table 5:** Factors associated with participants' knowledge and awareness regarding Alzheimer’s disease P: Pearson X2 test $: Exact probability test * P < 0.05 (significant)

Factors	Overall knowledge level				p-value
	Poor		Good		
	No	%	No	%	
Age in years					.152^$^
18-60	463	87.2%	68	12.8%	
> 60	14	100.0%	0	0.0%	
Gender					.499
Male	203	88.6%	26	11.4%	
Female	274	86.7%	42	13.3%	
Marital status					.049*
Single	259	85.2%	45	14.8%	
Married	187	91.7%	17	8.3%	
Divorced / widow	31	83.8%	6	16.2%	
Educational level					.227
Below secondary	12	100.0%	0	0.0%	
Secondary	112	88.2%	15	11.8%	
University	307	86.0%	50	14.0%	
Post-graduate	46	93.9%	3	6.1%	
Living status					.543
Alone	21	80.8%	5	19.2%	
Small family	281	87.5%	40	12.5%	
Big family	175	88.4%	23	11.6%	
Do you have a relative diagnosed with Alzheimer's disease?					.009*
Yes	78	79.6%	20	20.4%	
No	399	89.3%	48	10.7%	

## Discussion

This study aims to assess public awareness, knowledge, and attitude toward Alzheimer’s disease in Makkah, Saudi Arabia. We found that 87.5% of participants had poor knowledge, and with the rise in the prevalence of dementia and Alzheimer's disease among the continuously aging population, a focus is being placed on early detection and treatment. Difficulties diagnosing and managing mild cognitive impairment, which is usually the first presentation of dementia, are attributed to a triad of hesitant patients, unprepared healthcare providers, and environments of misconceptions and false beliefs [16]. However, Alzheimer's remains less known in the community than other chronic diseases among the elderly. There is a lack of factual information regarding Alzheimer's, and even professionals have misconceptions about its differences from normal aging [17]. People with Alzheimer's may also experience social stigma that delays their early diagnosis, optimal treatment, and effective delivery of quality care [18]. 

According to this study, 98 (18%) of the respondents had a relative with Alzheimer's disease, which is lower than a recent study which found 45% of the respondents had a relative diagnosed with Alzheimer's disease [19]. It is important to investigate the educational level of participants, particularly those who have a relative diagnosed with the disease. This is because it was reported previously that higher knowledge of patients' caregivers was associated with higher academic qualifications [20].

Regarding knowledge assessment, a number of studies have been conducted on Alzheimer's knowledge. Despite widespread knowledge of Alzheimer's, a systematic review found that most people do not understand the disease well. They think it is just part of the aging process [21]. A higher level of knowledge about Alzheimer's was found to be related to younger age, residing in an urban area, and having experience with Alzheimer's contact or care [22].

According to the analysis, the majority of 477 (87.5%) participants in this study had poor knowledge levels. Similarly, the highest proportion of participants (61%) in a previous study reported a low knowledge score [18]. Low knowledge was associated with older age and lower education. This is also compatible with another study where knowledge of Alzheimer's was modest and significantly associated with a family history of Alzheimer's and higher educational levels [23]. This gives an insight into the importance of addressing educational levels when investigating variable groups of participants. In this study, 48.3% know that genes are partially responsible for Alzheimer's disease. In contrast, a previous study reported that around 46% of participants believe that the cause of Alzheimer's disease is a brain disease [24]. In this study, the number of people who are aware that people with Alzheimer's disease are more likely to suffer from depression is 61.1%, while 27% oppose living in nursing homes. According to a previous study, more than half of the participants (56.2%) believed that nursing homes should not admit people with Alzheimer's disease [24].

Considering attitudes toward Alzheimer's disease patients, only 12.1% of this study feel embarrassed when a relative is diagnosed with the disease. To avoid embarrassing situations, 28.3% of participants believe it is better for diagnosed patients to avoid social events and life activities. This is supported by a previous study that revealed multiple emotional reactions to a person with Alzheimer's, ranging from anxiety and aggressiveness to prosocial reactions and rejection [25]. Similarly, a study by Marcinkiewicz and Reid found that a large proportion of the community perceived people with dementia as violent and aggressive [26]. In contrast, another study reported that participants believed that people with Alzheimer's disease should not be discriminated against [27]. Public interventions should address stigma and increase knowledge while understanding Alzheimer's sociocultural beliefs.

In a systematic review, stigmatizing perceptions and attitudes were mostly associated with modest disease knowledge, lack of contact experience, male gender, and younger age [28]. However, the relationship between knowledge and attitudes remains to be investigated.

In a previous study, scientific knowledge of Alzheimer's was not significantly related to personal fear of Alzheimer's, while social comfort was correlated negatively with it [29]. Developing a positive attitude and perception for patients with Alzheimer's disease does not necessarily require knowledge of Alzheimer's.

Strengths of the study

This study provides valuable evidence since there are a limited number of Saudi studies in this field. This study also included participants from a wide range of demographic and socio-economic backgrounds, making it easier for the Ministry of Health to focus on the targeted population to raise awareness about Alzheimer's disease. Furthermore, this study paved the way as the base for further studies in this aspect.

Limitations of the study

Limitations were present in the study. It may have been a highly selective group of cases because it was done in a specific setting. That the questionnaire was not culturally adopted. Therefore, generalizing the findings to Saudi Arabia's population may prove difficult. Statistical significance would also improve with a larger number of respondents.

## Conclusions

The results of this study revealed that the majority of 477 (87.5%) respondents had a poor knowledge level of awareness and knowledge of Alzheimer’s disease among the general population in Makkah. A significant association was reported between those with relatives diagnosed with Alzheimer's disease and good knowledge regarding the disease (P=.009). The general population should receive more attention since there is an increasing prevalence of Alzheimer's disease. Based on this study's findings and conclusions, we recommend (for the Ministry of Health) this topic be investigated serially and frequently to generate more evidence and data; (for the Ministry of Education and Health) convoys, medical missions, and conferences be held to increase the medical community's awareness of this topic; (for the Ministry of Media) media platforms and broadcasts are exploited in raising public awareness towards the significance and impact of Alzheimer's disease.

## Appendices

Alzheimer’s disease knowledge scale (ADKS) [15]

Demographic Characteristics

Age

-         <18

-         18-60

-         >60

Gender

-         Male

-         Female

Social status.

-         Single

-         Married

-         Divorced

-         Widow

-         Separated

Education level

-         Illiterate

-         Elementary

-         Intermediate

-         Secondary

-         Bachelor

-         Post-bachelor

Housing

-         Alone

-         Small family

-         Extended family

Awareness of Alzheimer’s Disease

Do you think that the loss of memory and forgetting names, appointments, and task repetition of questions are normal in the elderly and do not require medical consultation?

-       Agree

-       Disagree

Do you think that the change in the planning matters of everyday life and the difficulty of balancing the financial accounts is expected in the elderly?

-       Agree

-       Disagree

If one of your relatives was diagnosed with Alzheimer’s disease, do you prefer not to tell the person with illness?

-       Agree

-       Disagree

In the case of the patient diagnosed with Alzheimer’s, do you think that it is best to avoid going to social events and life activities in order to avoid embarrassment to the patient?

-       Agree

-       Disagree

Alzheimer’s disease may result from black magic or psychological distress or bad eye?

-       Agree

-       Disagree

In the case of the appearance of symptoms of dementia and memory disorder for a relative, will you resort to the popular types of alternative medicine?

-       Agree

-       Disagree

In case of difficulty in performing everyday tasks in a patient with Alzheimer’s, do you see it necessary to resort to the judiciary to save the patient's rights?

-       Agree

-       Disagree

Do you feel embarrassed if the diagnosis of your relative is Alzheimer’s disease?

-       Agree

-       Disagree

Do you tend to deny the diagnosis when one of your relatives has Alzheimer’s disease?

-       Agree

-       Disagree

Are you with the care of these patients in nursing homes by the state instead of keeping them at home?

-       Agree

-       Disagree

Knowledge of Alzheimer’s Disease

Risk factor:

It has been scientifically proven that mental exercise can prevent a person from getting Alzheimer’s disease.

-       Wrong

-       Right

-       Do not know

People in their 30s can have Alzheimer’s disease.

-       Wrong

-       Right

-       Do not know

Having high cholesterol may increase a person’s risk of developing Alzheimer’s disease.

-       Wrong

-       Right

-       Do not know

Prescription drugs that help delay progression of Alzheimer’s disease are available.

-       Wrong

-       Right

-       Do not know

Having high blood pressure may increase a person’s risk of developing Alzheimer’s disease.

-       Wrong

-       Right

-       Do not know

Genes can only partially account for the development of Alzheimer’s disease.

-       Wrong

-       Right

-       Do not know

Life impact:

People with Alzheimer’s disease are particularly prone to depression.

-       Wrong

-       Right

-       Do not know

Most people with Alzheimer’s disease live in nursing homes.

-       Wrong

-       Right

-       Do not know

It is safe for people with Alzheimer’s disease to drive, as long as they have a companion in the car at all times.

-       Wrong

-       Right

-       Do not know

Assessment and Diagnosis

When a person with Alzheimer’s disease becomes agitated, a medical assessment might reveal other health problems that caused the agitation.

-       Wrong

-       Right

-       Do not know

If trouble with memory and confusion started suddenly, it is likely due to Alzheimer’s disease.

-       Wrong

-       Right

-       Do not know

Symptoms of severe depression can be mistaken for symptoms of Alzheimer’s disease.

-       Wrong

-       Right

-       Do not know

Alzheimer’s disease is one type of dementia.

-       Wrong

-       Right

-       Do not know

Course

After symptoms of Alzheimer’s disease appear, the average life expectancy is 6 to 12 years.

-       Wrong

-       Right

-       Do not know

In rare cases, people have recovered from Alzheimer’s disease.

-       Wrong

-       Right

-       Do not know

A person with Alzheimer’s disease becomes increasingly likely to fall down as the disease gets worse.

-       Wrong

-       Right

-       Do not know

Eventually, a person with Alzheimer’s disease will need 24-h supervision.

-       Wrong

-       Right

-       Do not know

Caregiving

People with Alzheimer’s disease do best with simple instructions given one step at a time.

-       Wrong

-       Right

-       Do not know

When people with Alzheimer’s disease begin to have difficulty taking care of themselves, caregivers should take over right away.

-       Wrong

-       Right

-       Do not know

If a person with Alzheimer’s disease becomes alert and agitated at night, a good strategy is to try to make sure that the person gets plenty of physical activity during the day.

-       Wrong

-       Right

-       Do not know

When people with Alzheimer’s disease repeat the same question or story several times, it is helpful to remind them that they are repeating themselves.

-       Wrong

-       Right

-       Do not know

Once people have Alzheimer’s disease, they are no longer capable of making informed decisions about their own care.

-       Wrong

-       Right

-       Do not know

Treatment

People whose Alzheimer’s disease is not yet severe can benefit from psychotherapy for depression and anxiety.

-       Wrong

-       Right

-       Do not know

Poor nutrition can make the symptoms of Alzheimer’s disease worse.

-       Wrong

-       Right

-       Do not know

When a person has Alzheimer’s disease, using reminder notes can contribute to decline.

-       Wrong

-       Right

-       Do not know

Alzheimer’s disease cannot be cured.

-       Wrong

-       Right

-       Do not know

Symptoms

Tremor or shaking of the hands or arms is a common symptom in people with Alzheimer’s disease.

-       Wrong

-       Right

-       Do not know

Trouble handling money or paying bills is a common early symptom of Alzheimer’s disease.

-       Wrong

-       Right

-       Do not know

One symptom that can occur with Alzheimer’s disease is believing that other people are stealing one’s things.

-       Wrong

-       Right

-       Do not know

Most people with Alzheimer’s disease remember recent events better than things that happened in the past.

-       Wrong

-       Right

-       Do not know

## References

[1] DeTure MA, Dickson DW (2019). The neuropathological diagnosis of Alzheimer's disease. Mol Neurodegener.

[2] (2022). Saudi Alzheimer's disease association. https://alz.org.sa.

[3] Breijyeh Z, Karaman R (2020). Comprehensive review on Alzheimer’s disease: causes and treatment. Molecules.

[4] Connell CM, Scott Roberts J, McLaughlin SJ, Akinleye D (2009). Racial differences in knowledge and beliefs about Alzheimer disease. Alzheimer Dis Assoc Disord.

[5] Connell CM, Scott Roberts J, McLaughlin SJ (2007). Public opinion about Alzheimer disease among blacks, Hispanics, and whites: results from a national survey. Alzheimer Dis Assoc Disord.

[6] Tsolaki M, Paraskevi S, Degleris N, Karamavrou S (2009). Attitudes and perceptions regarding Alzheimer's disease in Greece. Am J Alzheimers Dis Other Demen.

[7] Dilworth-Anderson P, Pierre G, Hilliard TS (2012). Social justice, health disparities, and culture in the care of the elderly. J Law Med Ethics.

[8] Pope SK, Shue VM, Beck C (2003). Will a healthy lifestyle help prevent Alzheimer's disease?. Annu Rev Public Health.

[9] Solfrizzi V, Capurso C, D'Introno A (2008). Lifestyle-related factors in predementia and dementia syndromes. Expert Rev Neurother.

[10] Erickson KI, Weinstein AM, Lopez OL (2012). Physical activity, brain plasticity, and Alzheimer's disease. Arch Med Res.

[11] Defina LF, Willis BL, Radford NB (2013). The association between midlife cardiorespiratory fitness levels and later-life dementia: a cohort study. Ann Intern Med.

[12] Hötting K, Röder B (2013). Beneficial effects of physical exercise on neuroplasticity and cognition. Neurosci Biobehav Rev.

[13] Vassallo N, Scerri C (2013). Mediterranean diet and dementia of the Alzheimer type. Curr Aging Sci.

[14] Alhazzani AA, Alqahtani AM, Alqahtani MS, Alahmari TM, Zarbah AA (2020). Public awareness, knowledge, and attitude toward Alzheimer’s disease in Aseer region, Saudi Arabia. Egypt J Neurol Psychiatr Neurosurg.

[15] Carpenter BD, Balsis S, Otilingam PG, Hanson PK, Gatz M (2009). The Alzheimer's Disease Knowledge Scale: development and psychometric properties. Gerontologist.

[16] Lu Y, Liu C, Wells Y, Yu D (2022). Challenges in detecting and managing mild cognitive impairment in primary care: a focus group study in Shanghai, China. BMJ Open.

[17] Umegaki H, Suzuki Y, Ohnishi J, Iguchi A (2009). Changes in the perception of dementia in Japan. Int Psychogeriatr.

[18] Mahoney DF, Cloutterbuck J, Neary S, Zhan L (2005). African American, Chinese, and Latino family caregivers' impressions of the onset and diagnosis of dementia: cross-cultural similarities and differences. Gerontologist.

[19] McParland P, Devine P, Innes A, Gayle V (2012). Dementia knowledge and attitudes of the general public in Northern Ireland: an analysis of national survey data. Int Psychogeriatr.

[20] Alorfi NM (2022). Public awareness of Alzheimer’s disease: a cross-sectional study from Saudi Arabia. Int J Gen Med.

[21] Cahill S, Pierce M, Werner P, Darley A, Bobersky A (2015). A systematic review of the public's knowledge and understanding of Alzheimer's disease and dementia. Alzheimer Dis Assoc Disord.

[22] Rosato M, Leavey G, Cooper J, De Cock P, Devine P (2019). Factors associated with public knowledge of and attitudes to dementia: A cross-sectional study. PLoS One.

[23] Hamieh N, Sharara E, Salibi N, Mrad P, Chaaya M (2019). Public knowledge of, perceptions about and attitudes towards dementia: a cross-sectional survey among Lebanese primary health care attenders. Community Ment Health J.

[24] Algahtani H, Shirah B, Alhazmi A, Alshareef A, Bajunaid M, Samman A (2020). Perception and attitude of the general population towards Alzheimer's disease in Jeddah, Saudi Arabia. Acta Neurol Belg.

[25] Cohen M, Werner P, Azaiza F (2009). Emotional reactions of Arab lay persons to a person with Alzheimer's disease. Aging Ment Health.

[26] Marcinkiewicz A, Reid S (2016). Attitudes to dementia findings from the 2015 British Social Attitudes Survey. NatCen Social.

[27] Yang HF, Cong JY, Zang XY, Jiang N, Zhao Y (2015). A study on knowledge, attitudes and health behaviours regarding Alzheimer's disease among community residents in Tianjin, China. J Psychiatr Ment Health Nurs.

[28] Herrmann LK, Welter E, Leverenz J, Lerner AJ, Udelson N, Kanetsky C, Sajatovic M (2018). A systematic review of dementia-related stigma research: can we move the stigma dial?. Am J Geriatr Psychiatry.

[29] Chang CY, Hsu HC (2020). Relationship between knowledge and types of attitudes towards people living with dementia. Int J Environ Res Public Health.

